# Geniposide Ameliorated Dexamethasone-Induced Cholesterol Accumulation in Osteoblasts by Mediating the GLP-1R/ABCA1 Axis

**DOI:** 10.3390/cells10123424

**Published:** 2021-12-06

**Authors:** Yizhou Zheng, Yaosheng Xiao, Di Zhang, Shanshan Zhang, Jing Ouyang, Linfu Li, Weimei Shi, Rui Zhang, Hai Liu, Qi Jin, Zhixi Chen, Daohua Xu, Longhuo Wu

**Affiliations:** 1 College of Pharmacy, Gannan Medical University, Ganzhou 341000, China; yizzheng@gmu.edu.cn (Y.Z.); shanszhang@gmu.edu.cn (S.Z.); lflfllf2001@gmu.edu.cn (L.L.); wm_shi@gmu.edu.cn (W.S.); ruizhang@gmu.edu.cn (R.Z.); hailiu@gmu.edu.cn (H.L.); jinqimy@163.com (Q.J.); czxb22@163.com (Z.C.); 2 Department of Orthopedics, The First Affiliated Hospital of Gannan Medical University, Ganzhou 341000, China; yaosxiao@gmu.edu.cn; 3 Department of Medical Imaging, The First Affiliated Hospital of Gannan Medical University, Ganzhou 341000, China; dizhang@gmu.edu.cn; 4 College of Rehabilitation, Gannan Medical University, Ganzhou 341000, China; jingouy@gmu.edu.cn; 5 Key Laboratory of Traditional Chinese Medicine and New Pharmacy Development, Guangdong Medical University, Dongguan 523808, China; daohuaxu@gdmu.edu.cn

**Keywords:** osteoporosis, glucocorticoid, cholesterol, geniposide, GLP-1R, ABCA1

## Abstract

Background: Overexposure to glucocorticoid (GC) produces various clinical complications, including osteoporosis (OP), dyslipidemia, and hypercholesterolemia. Geniposide (GEN) is a natural iridoid compound isolated from Eucommia ulmoides. Our previous study found that GEN could alleviate dexamethasone (DEX)-induced differentiation inhibition of MC3T3-E1 cells. However, whether GEN protected against Dex-induced cholesterol accumulation in osteoblasts was still unclear. Methods: DEX was used to induce rat OP. Micro-CT data was obtained. The ALP activity and mineralization were determined by the staining assays, and the total intracellular cholesterol was determined by the ELISA kits. The protein expression was detected by western blot. Results: GEN ameliorated Dex-induced micro-structure damages and cell differentiation inhibition in the bone trabecula in rats. In MC3T3-E1 cells, Dex enhanced the total intracellular cholesterol, which reduced the activity of cell proliferation and differentiation. Effectively, GEN decreased DEX-induced cholesterol accumulation, enhanced cell differentiation, and upregulated the expression of the GLP-1R/ABCA1 axis. In addition, inhibition of ABAC1 expression reversed the actions of GEN. Treatment with Exendin9-39, a GLP-1R inhibitor, could abrogate the protective activity of GEN. Conclusions: GEN ameliorated Dex-induced accumulation of cholesterol and inhibition of cell differentiation by mediating the GLP-1R/ABCA1 axis in MC3T3-E1 cells.

## 1. Introduction

Osteoporosis (OP) is a progressive systemic skeletal disease, which has been documented by the World Health Organization. It is characterized by low bone mass and deterioration of bone micro-architecture, and these pathological changes can cause an increase in bone tissue fragility and susceptibility to fracture [1]. OP is also related to a pathological imbalance featured by increased osteoclastic activity and decreased osteoblastic activity [2]. Glucocorticoid-induced OP (GIOP) is a clinically common cause of secondary OP [3]. Chronic exposure to glucocorticoid (GC) induces a decline in bone strength, a decrease in osteoblastogenesis and osteoblasts, and an increase in osteocyte apoptosis. The effects of GC on osteoblasts differentiation might be regulated by signaling pathways, such as Wnt, BMP-Runx2, and PPAR-γ2 pathways [4]. However, the underlying mechanisms of GC in regulating the development of OP are still unclear.

A retrospective study has been shown that dyslipidemia in postmenopausal women is positively associated with OP progression [5]. Furthermore, hypercholesterolemia or high-cholesterol diets have been reported to reduce bone strength and enhance the potential risks of bone fracture and OP [6]. Excessive cholesterol in the body may cause OP [7]. Statins, inhibitors of HMG-CoA reductase to block cholesterol synthesis, have been shown to increase bone formation, bone mineral density, and osteoblasts differentiation [8]. In addition, stimulation by cholesterol with different concentrations has been reported to inhibit the proliferation and the differentiation of osteoblasts [9]. It can be postulated that intracellular cholesterol accumulation exhibits adverse effects on the biological functions of osteoblasts. However, the underlying mechanism of cholesterol in OP development still needs more investigation.

Geniposide (GEN), a purified iridoid glycoside from the Eucommia, has various pharmacological activities, including anti-diabetes, anti-inflammation, anti-cancer, and neuroprotection [10,11,12]. Recently, GEN has been shown to increase osteoblasts’ proliferation and differentiation by activating the Wnt/β-catenin signaling pathway [13]. GEN can enhance insulin secretion by activating the expression of glucagon-like peptide 1 receptor (GLP-1R) in INS-1 cells [14]. ATP-binding cassette transporter A1 (ABCA1) is a carrier for the intracellular cholesterol out of the cytoplasm [15]. Exendin-4, a GLP-1R agonist, induces the expression of ABCA1 in glomerular endothelial cells [16]. Our previous study found that GEN could ameliorate dexamethasone (DEX)-induced inhibition of osteoblast proliferation and differentiation by activating the expression of GLP-1R [17]. This article will investigate whether GEN exhibits protective effects against cholesterol accumulation by mediating ABCA1 expression.

## 2. Materials and Methods

### 2.1. General

This project (GMU202011) was approved by the Institutional Animal Care and Use Committee of Gannan Medical University, according to the Declaration of Helsinki Principles. The male rats were six-week-old and treated with an adaptive feeding for a week in an SPF-grade room, according to the guideline of the central animal care. Water and food were freely accessible, and a circumstance with a 12 h light/dark cycle (Temperature: 21–23 °C; humidity: 45–55%) was provided.

### 2.2. Rat OP Models

A total of 48 rats (200 ± 20 g) were randomly divided into four groups. Each group included six rats, and the animal experiments were repeated twice. These groups contained: (I) negative control (NC) group, only treated with vehicle; (II) DEX group, Dexamethasone sodium phosphate (Tianjian Pharmaceutical Group Xinzheng Co. Ltd, Tianjin, China) at the dose of 5 mg/kg (twice/week) were intramuscularly injected into the gluteus maximus of rats; (III) DEX plus GEN (50 mg/kg/day); (IV) DEX plus GEN (100 mg/kg/day). The rats in the treated groups were intragastrically administered with GEN (50 mg/kg and 100 mg/kg, respectively) [18]. No obvious adverse reactions were observed, and no rats died during the treatment. The animal experimental procedures were in line with the requirements of the European Union directive 2010/63/EU. After 16 weeks, rats were sacrificed, and the integral right femurs were collected. Some femurs were put in phosphate-buffered saline (PBS, Sigma, St. Louis, MO, USA)-moistened gauzes and prepared for micro-CT scanning. The remained femurs were kept in a −80 °C refrigerator for histochemical examination and immunohistochemical evaluation.

### 2.3. Micro-Computed Tomography Scanning

The proximal femurs were scanned ex vivo using μCT (Bruker-microCT SkyScan 1176, Kontich, Belgium). The parameters were set as standard, mainly including the voltage of 65 kV, current of 385 mA, and resolution of 10.44. All images were reconstructed by NRecon software (version 1.13, Bruker-microCT, SkyScan, Kontich, Belgium). In the bone marrow cavity of the rat distal femur, the bone trabecula below the growth plate within the range of 0.5–1.0 mm was set as the region of interest 1 (ROI1), and the bone morphology index and the bone mineral density (BMD) were measured. The cortical bone below the growth plate within the range of 4.0–4.5 mm was regarded as the region of interest 2 (ROI2). The trabecular morphometric indices included trabecular bone thickness (Tb.Th), trabecular bone separation (Tb.Sp), bone surface area (BS), bone volume (BV), and bone separation (SMI).

### 2.4. Cell Culture

MC3T3-E1 cell line was purchased from the Chinese Academy of Sciences Cell Bank. Cells were cultured in Modified Eagle’s Medium of Alpha (α-MEM) (Gibco) containing 10% fetal bovine serum (FBS) (Gibco) and 100 U penicillin/streptomycin (Gibco). For developing osteoblasts differentiation, the mixed medium above was supplemented with 10 mM β-glycerophosphate and 50 μg/mL ascorbic acid, and then the osteogenic induction medium (OIM) was prepared. After 15-day incubation with OIM, MC3T3-E1 cells were used for further investigation.

### 2.5. Mineralization Assays

After removing the medium, cells were washed with PBS three times. Then, cells were fixed with 80% ethanol for 15 min and subsequently stained with 0.5% Alizarin Red S (Sigma, St Louis, MO, USA) for 20 min at 37 °C. Finally, the optical density was detected at the wavelength of 550 nm. The orange-red parts represented the positive position, and the color intensity indicated the degree of calcium deposition.

### 2.6. ALP Staining

The plates were added 80% ethanol to soak osteoblasts for 15 min and equilibrated with ALP buffer (0.15 M NaCl, 0.15 M Tris-HCl, and 1 mM MgCl2, pH 9.5) for 15 min. Next, the plates were stained with BCIP/NBT solution (Sigma, St Louis, MO, USA) for 1 h at 37 °C, and then washed with deionized water three times. The ALP activity was detected by a microplate reader (Thermo Fisher Scientific Inc., Waltham, MA, USA) at the wavelength of 520 nm. The blue parts represented the active position of ALP.

### 2.7. Concentration Detection of the Total Intracellular Cholesterol

At the confluence of 50%, the osteoblasts were incubated with DEX and/or GEN for 15 days. For the sample collection, the osteoblasts were digested by trypsin (Solarbio, Beijing, China) and then centrifuged at 3000× *g* rpm for 1 min at 4 °C. The sedimented cells were shaken into a uniform turbid liquid and divided equally into two parts for the concentration detection of cholesterol and protein, respectively. To detect the total intracellular cholesterol, isopropanol (Vsample:Visopropanol = 1:9) was added, and then the mixture was centrifuged at 12,000× *g* rpm for 15 min at 4 °C. The supernatant was collected and diluted by 1:2000. Then, it was prepared for detecting the concentration of cholesterol in osteoblasts by the mouse cholesterol ELISA kit (Cat.no. K4440-100, BioVision, Inc. Milpitas, CA, USA). The concentration of cholesterol was normalized to that of the total protein.

### 2.8. MTT Assays

MC3T3-E1 cells (5 × 10^3^ cells/well) were cultured with α-MEM (Gibco) containing 10% FBS (Gibco) and 100 U penicillin/streptomycin (Gibco) and OIM for 15 days at 37 °C. The different concentrations of cholesterol (1, 12.5, 25, 50, and 100 μM) (Sigma, St Louis, MO, USA) were added and then co-incubated for 3 days. The assays were conducted according to the kit instructions (Beyotime, Shanghai, China). Specifically, MTT (0.5 mg/mL) was added and co-incubated with cells for 4 h at 37 °C. Then, 150 μL DMSO was added to dissolve the formazan in the dark. The wavelength of 490 nm was used for measurement using the microplate reader (Thermo Fisher Scientific Inc., Waltham, MA, USA).

### 2.9. Western Blot

The total proteins in the osteoblasts were extracted and the protein concentrations were determined by the BCA protein assay kit (Beyotime, Shanghai, China). 30 μg of the total proteins were subjected to 10% SDS-PAGE and then transferred onto PVDF membranes. After being blocked in TBS containing 5% skimmed milk for 1 h, the membranes were co-incubated with the primary antibodies at 4 °C overnight against RUNX2 (1:1000 dilution; MyBioSource, cat.no.127554, San Diego, CA, USA), OPN (1:1000 dilution; Proteintech, cat.no.22952-1-AP, Rosemont, IL, USA), GLP-1R (1:1000 dilution; ABGENT, cat.no.AP52040), ABCA1 (1:1000 dilution; Affinity, cat.no.DF8233), apoA-I (1:1000 dilution; Affinity, cat.no.DF6264), and GAPDH (1:1000 dilution; Affinity, cat.no.AF7021), and then with HRP-labeled goat anti-rabbit secondary antibody (1:5000 dilution; Boster Biological Technology, Wuhan, China). Protein bands were detected using the enhanced chemiluminescence detection system and quantified using the Fiji ImageJ (version 1.51r; NIH, Bethesda, MD, USA).

### 2.10. Statistical Analysis

All experiments were implemented separately in triplicate and data are presented as mean ± standard deviation (SD). GraphPad Prism 7 Software (GraphPad Software Inc., La Jolla, CA, USA) was applied to perform statistical analysis. The statistical significance was detected via one-way analysis of variance (ANOVA) prior to Bonferroni’s multiple comparisons test. A value of *p* < 0.05 represented a statistically significant difference.

## 3. Results

### 3.1. GEN Protected against DEX-Induced OP in Rat Models

**Figure 1:**
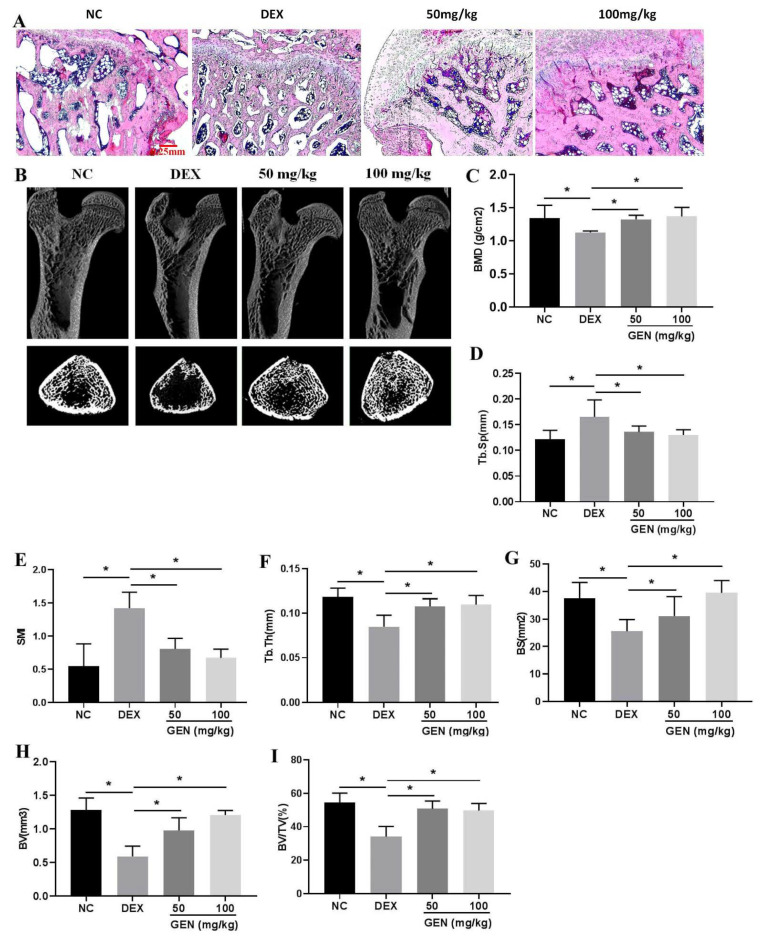
GEN protected against DEX-induced OP in rat models (*n* = 6, repeated twice). (**A**) The HE staining of the bone trabecula on the femoral neck of the proximal femur (×40 magnification). (**B**) 3D reconstruction and 2D reconstruction of micro-CT on the proximal femur. (**C**) Bone mineral density (BMD) of the proximal femur. The values of Tb.Sp (**D**), SMI (**E**), Tb.Th (**F**), BS (**G**), BV (**H**), and BV/TV (**I**) were detected. All experiments were implemented separately in triplicate. * *p* < 0.05. NC, negative control; 50 mg/kg, Dex + 50 mg/kg GEN; 100 mg/kg, Dex + 100 mg/kg GEN.

Chronic exposure to DEX could induce damages to the bone system. In DEX-induced rat models, the disordered and thinner bone trabecula was observed by the HE staining assays, compared with that in the NC group (Figure 1A). Data from micro-CT in the proximal femur showed that DEX induced the loss of the bone trabecula (Figure 1B), accompanied by decreased BMD (Figure 1C), increased indices of Tb.Sp (Figure 1D) and SMI (Figure 1E), and decreased indices of Tb.Th (Figure 1F), BS (Figure 1G), BV (Figure 1H), and BV/TV (Figure 1I). Treatment with GEN (50 mg/kg and 100 mg/kg, respectively) could significantly reverse DEX-induced loss of the bone trabecula, indicating the protective activity of GEN in the therapeutic management of OP.

### 3.2. GEN Ameliorated DEX-Induced Inhibition of Osteoblast Differentiation

**Figure 2:**
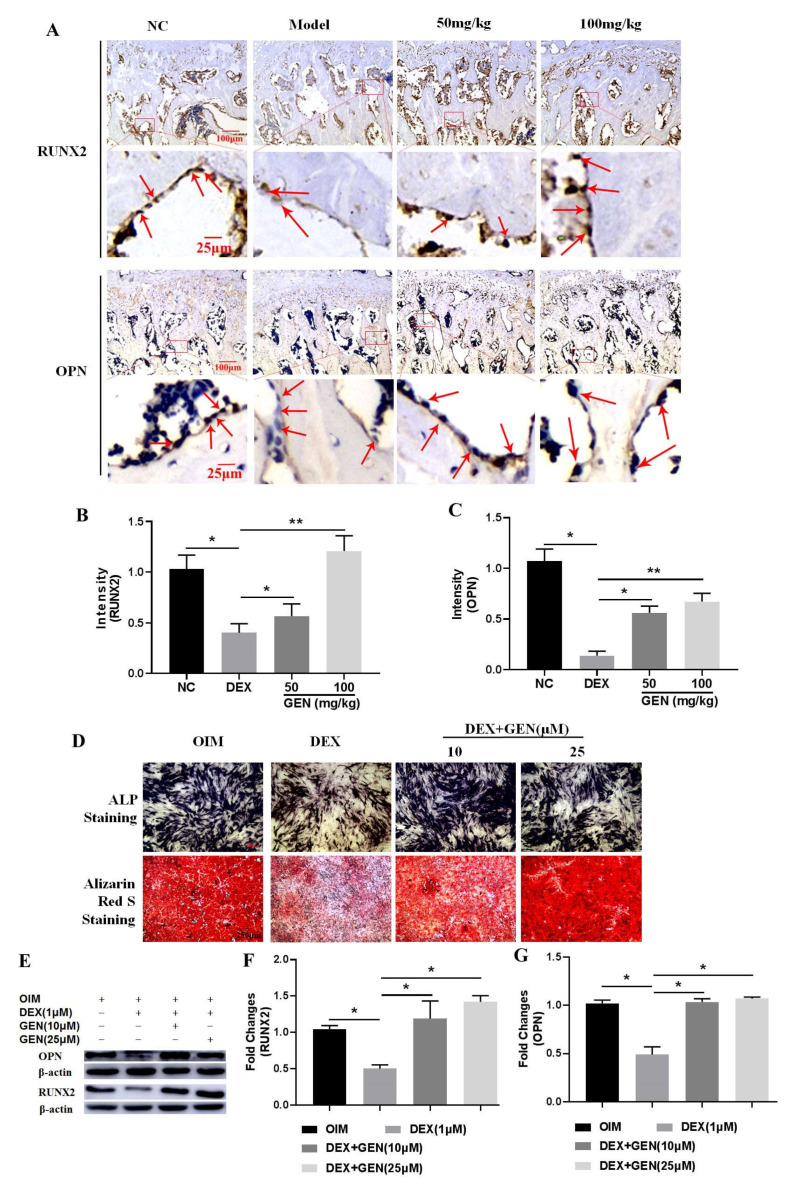
GEN ameliorated DEX-induced inhibition of osteoblast differentiation. (**A**) The immunohistochemical study of RUNX2 and OPN in the bone trabecula of the rat proximal femurs (×40 magnification). The staining intensity of RUNX2 (**B**) and OPN (**C**) was evaluated. (**D**) The ALP staining and the Alizarin Red S staining assays were conducted (×100 magnification). The protein expression of RUNX2 (**E**,**F**) and OPN (**E**,**G**) were determined by western blot. All experiments were implemented separately in triplicate. * *p* < 0.05; ** *p* < 0.01. NC, negative control; 50 mg/kg, Dex + 50 mg/kg GEN; 100 mg/kg, Dex + 100 mg/kg GEN.

To further explore the potential mechanism of GEN in protecting against DEX-induced loss of the bone trabecula, the differentiation activity of osteoblasts was detected. The in vivo immunohistochemical examination showed that DEX could decrease the expression of RUNX2 and OPN in the bone trabecula of the proximal femurs (Figure 2A–C). In vitro study, MC3T3-E1 cells were stimulated with DEX (1 μM). The ALP staining assays showed that DEX significantly decreased ALP activity and mineralization (Figure 2D), indicating suppression of cell differentiation. Consistently, the protein expression of RUNX2 (Figure 2E,F) and OPN (Figure 2E,G) were also attenuated by treatment with DEX in MC3T3-E1 cells. The doses of GEN (10 μM and 25 μM) used in vitro study were determined as those in our previous study [17]. GEN could effectively ameliorate DEX-induced inhibition of osteoblast differentiation in vivo and in vitro. Collectively, GEN protected against DEX-induced loss of the bone trabecula by promoting osteoblast differentiation.

### 3.3. GEN Ameliorated DEX-Induced Cholesterol Accumulation in MC3T3-E1 Cells

**Figure 3:**
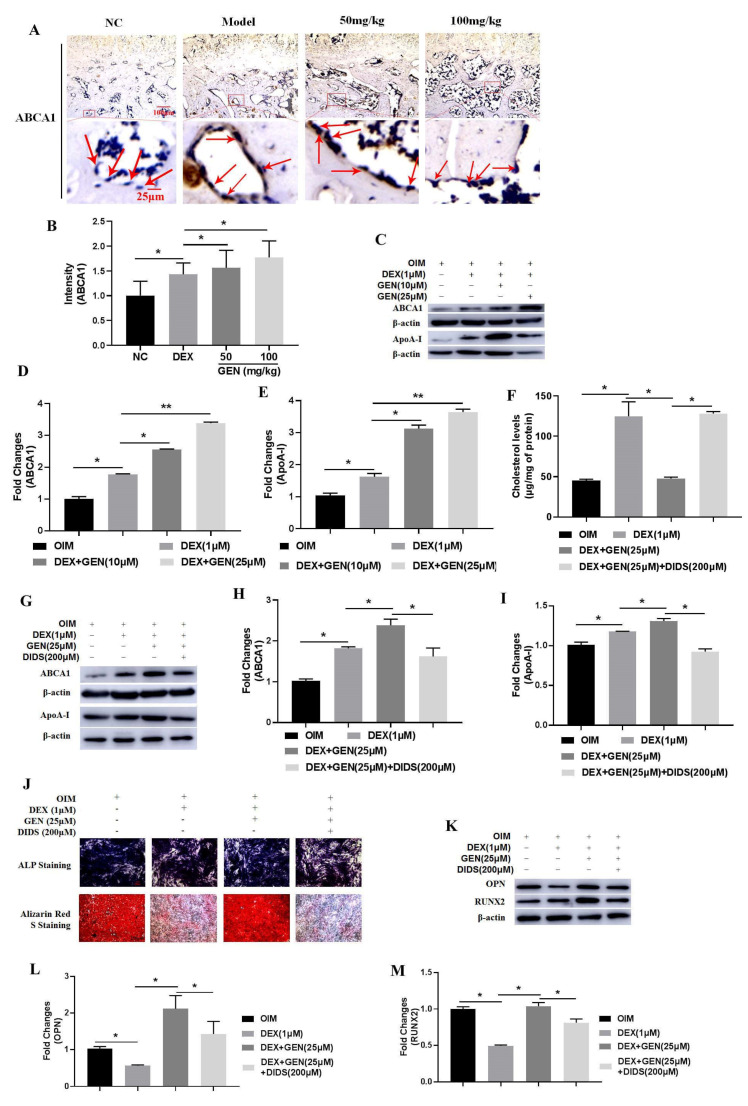
GEN ameliorated DEX-induced cholesterol accumulation in MC3T3-E1 cells. (**A**) MTT assays were conducted to investigate the effects of cholesterol on cell viability. (**B**) The level of intracellular cholesterol after administration with exogenous cholesterol. (**C**–**E**) The protein expression of RUNX2 and OPN in CHO (50 μM)-treated MC3T3-E1 cells was determined by western blot. (**F**) The level of the total intracellular cholesterol was measured by the ELISA kit. All experiments were implemented separately in triplicate. * *p* < 0.05; ** *p* < 0.01. OIM, osteogenic induction medium; CHO, cholesterol.

Cholesterol stimulation has been demonstrated to exhibit adverse effects on osteoblast differentiation [9]. In this study, the biological effects of cholesterol on osteoblast differentiation were also investigated. MTT assay was conducted to explore the effects of cholesterol on cell viability (Figure 3A). The results showed that cholesterol could significantly decrease cell viability in a dose-dependent manner. At the dose of 50 μM, cholesterol exhibited almost the strongest inhibition on the cell viability. Exogenous cholesterol could effectively increase the levels of intracellular cholesterol (Figure 3B). In addition, cholesterol (50 μM) showed the inhibitory activity on the expression of RUNX2 (Figure 3C,D) and OPN (Figure 3C,E) in MC3T3-E1 cells. Then, the effects of GEN on DEX-induced cholesterol accumulation in MC3T3-E1 cells were investigated. The level of the total intracellular cholesterol was detected (Figure 3F). It was consistently shown that DEX increased the accumulation of cholesterol. Treatment with GEN might reverse the effects of DEX, decreasing cholesterol accumulation. Thus, GEN ameliorated Dex-induced accumulation of cholesterol, which produced adverse effects on the differentiation of MC3T3-E1 cells.

### 3.4. GEN Ameliorated DEX-Induced Cholesterol Accumulation by Increasing ABCA1 Expression

**Figure 4:**
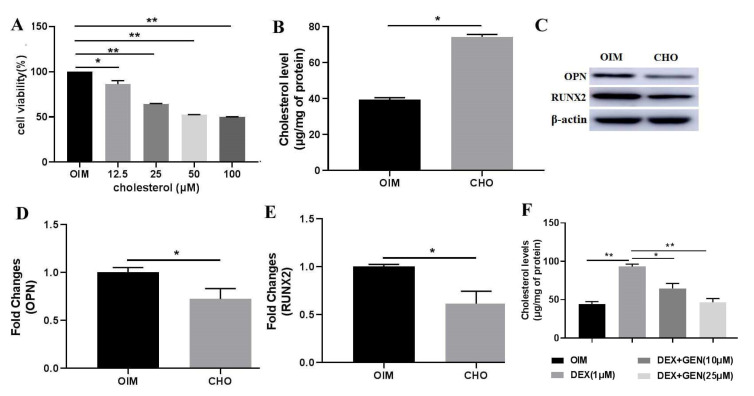
GEN ameliorated DEX-induced cholesterol accumulation by increasing ABCA1 expression. (**A**,**B**) The in vivo immunohistochemical examination of ABCA1 expression was conducted. (**C**–**E**) The protein expression of ABCA1 and ApoA-I was detected by western blot in DEX-treated MC3T3-E1 cells. (**F**) The level of the total intracellular cholesterol was measured using the ELISA kit in MC3T3-E1 cells co-treated with DEX and DIDS (200 μM). (**G**–**I**) The protein expression of ABCA1 and ApoA-I was detected by western blot in DEX/DIDS-treated MC3T3-E1 cells. (**J**) The ALP staining and the Alizarin Red S staining assays were conducted (×100 magnification). (**K**–**M**) The protein expression of RUNX2 and OPN was detected by western blot in DEX/DIDS-treated MC3T3-E1 cells. All experiments were implemented separately in triplicate. * *p* < 0.05; ** *p* < 0.01. OIM, osteogenic induction medium. NC, negative control; 50 mg/kg, Dex + 50 mg/kg GEN; 100 mg/kg, Dex + 100 mg/kg GEN.

From the study in vivo, we found that the expression of ABCA1 was up-regulated by DEX through the immunohistochemical assays (Figure 4A,B). In DEX-treated MC3T3-E1 cells, the protein expression of ABCA1 and ApoA-I was also up-regulated slightly (Figure 4C–E). Interestingly, treatment with GEN could further enhance the expression of ABCA1 and ApoA-I in vivo and in vitro. To further explore the roles of ABCA1 in GEN-mediated protection against the negative effects of cholesterol accumulation on cell differentiation, the ABCA1 antagonist DIDS (200 μM) was added. As expected, the level of the total intracellular cholesterol was significantly increased (Figure 4F), and the expression of ABCA1 and ApoA-I was decreased (Figure 4G–I), abrogating the effects of GEN. In addition, The GEN-enhanced ALP activity (Figure 4J), mineralization (Figure 4J), and RUNX2 and OPN expression (Figure 4K–M) were also decreased by DIDS. Collectively, GEN ameliorated DEX-induced cholesterol accumulation by increasing ABCA1 expression.

### 3.5. GEN Promoted ABCA1-Mediated Cholesterol Metabolism by Activating GLP-1R

**Figure 5:**
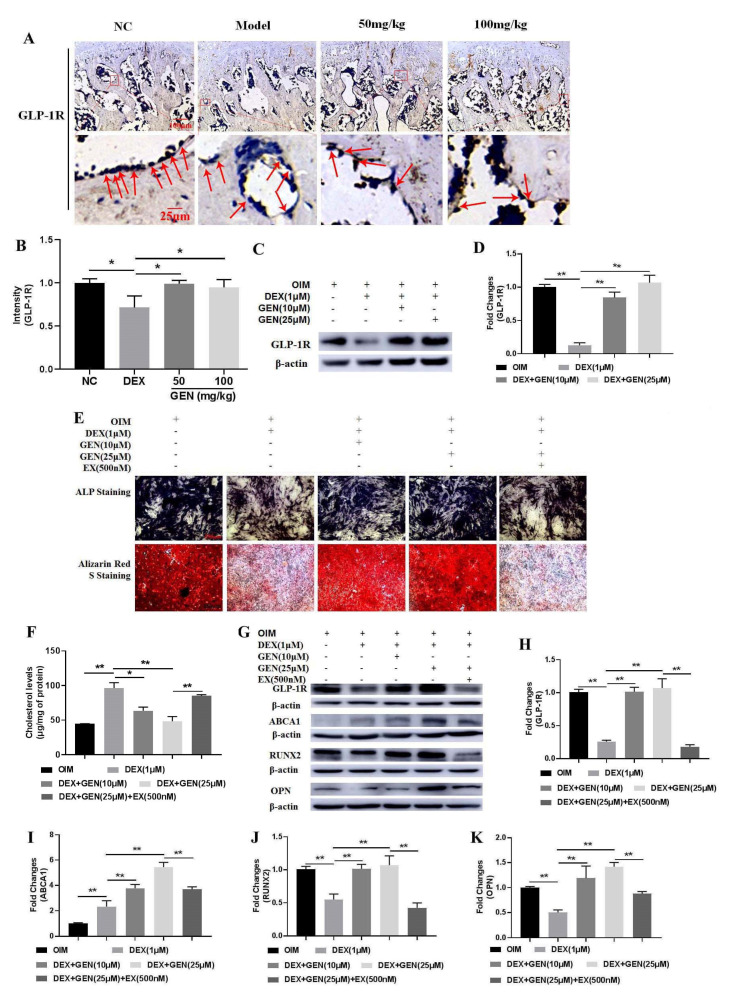
GEN promoted ABCA1-mediated cholesterol metabolism in a GLP-1R-dependent manner. (**A**,**B**) The in vivo immunohistochemical examination of GLP-1R expression was conducted. (**C**,**D**) The protein expression of GLP-1R was determined by western blot in DEX-treated MC3T3-E1 cells. (**E**) The ALP staining and the Alizarin Red S staining assay were performed in EX-treated MC3T3-E1 cells (×100 magnification). (**F**) The level of the total intracellular cholesterol was measured using the ELISA kit. (**G**–**K**) The protein expression of GLP-1R, ABCA1, RUNX2, and OPN was detected by western blot. All experiments were implemented separately in triplicate. * *p* < 0.05; ** *p* < 0.01. NC, negative control; 50 mg/kg, Dex + 50 mg/kg GEN; 100 mg/kg, Dex + 100 mg/kg GEN; EX, Exendin9-39.

To further investigate the potential mechanism of GEN in promoting ABCA1-mediated cholesterol metabolism, the expression of GLP-1R was explored. As found in Figure 5A,B, GEN could significantly up-regulate the expression of GLP-1R in vivo. Consistently, the expression of GLP-1R was also increased by GEN in DEX-treated MC3T3-E1 cells (Figure 5C,D). To further explore the roles of GLP-1R, a competitive antagonist, Exendin9-39 (EX), was used. In DEX-treated MC3T3-E1 cells, EX could abrogate GEN-ameliorated total intracellular cholesterol (Figure 5F). In addition, EX also attenuated the effects of GEN on the activity of ALP and mineralization (Figure 5E) and the expression of GLP-1R (Figure 5G,H), ABCA1 (Figure 5G–I), RUNX2 (Figure 5G–J), and OPN (Figure 5G–K). Collectively, GEN promoted ABCA1-mediated cholesterol metabolism in a GLP-1R-dependent manner.

## 4. Discussion

Metabolic syndrome (MetS) has been associated with insulin resistance, obesity, hypertension, and dyslipidemia. Recently, a group of studies has been demonstrated the roles of MetS in the pathological development of OP [19,20]. Analysis of data from the National Health and Nutrition Examination Survey (NHANES) database shows that the levels of high-density lipoprotein cholesterol are negatively associated with BMD, particularly in females [21]. The important roles of lipid metabolism in bones have been reported. However, the potential regulatory mechanism of cholesterol in bone is still unclear. In this study, we found that DEX could induce the development of OP by promoting cholesterol accumulation in MC3T3-E1 cells. The possible mechanism might be associated with the inhibitory effects of DEX on cell differentiation by increasing the accumulation of intracellular cholesterol. GEN, a naturally occurring iridoid glycoside, could effectively ameliorate the effects against DEX-induced cholesterol accumulation by activating the expression of the GLP-1R/ABCA1 axis in MC3T3-E1 cells.

Lipids play a critical role in bone metabolism. For instance, lipids on the plasma membrane can transduce a cascade of signals. However, excessive lipid accumulation in osteocytes dramatically influences bone quality, as found by deceased BMD, increased osteoclast resorption, and decreased osteoblastic formation [22]. Targeting lipid metabolism with lipid-lowering drugs statins can significantly improve BMD and ameliorate the negative phenotypes [23]. Cholesterol, a significant component of lipid rafts, is involved in bone metabolism [24]. Fenofibrate is a PPARα agonist used for lipid-lowering to reduce the levels of cholesterol. It has been shown that fenofibrate promotes osteoblast differentiation by increasing the expression of BMP2 in MC3T3-E1 cells [25]. Consistently, the cholesterol-lowering agent lovastatin has been reported to increase osteogenic differentiation [26]. A study is reported that cholesterol treatment induces inhibitory effects on osteoblastic differentiation in mouse mesenchymal stem cells (MSC). Our study showed that exogenous cholesterol could decrease the osteogenic differentiation in MC3T3-E1 cells, as indicated by the down-regulation of RUNX2 and OPN expression.

As the secondary OP, GIOP accounts for 20% of osteoporosis diagnoses. Exposure to GCs for more than 6 months may result in OP in 50% of patients [27]. At the physiological concentration, endogenous GCs are required for the maintenance of osteoblasts homeostasis. At the therapeutic concentration, exogenous GCs have been reported to decrease the formation and survival of osteoblasts. Specifically, GCs attenuate the expression of RUNX2 and inhibit osteoblast maturation by mediating BMP-2 and Wnt/β-catenin signaling pathways [28]. GIOP has been associated with increased bone marrow adiposity. Compared with bone marrow MSC (bmMSC), MSC from the proximal end of the femur (pfMSC) is more vulnerable to producing lipid droplet accumulation, leading to osteoporotic fractures [29]. However, GCs at the pharmacological concentration alone cannot induce lipid accumulation but lead to significant cell cytotoxicity and apoptosis in pfMSC [29]. In another study, it shows that GCs significantly reduce the proliferative activity of pfMSC [30]. DEX can promote lipid accumulation, increase adipogenesis, and inhibit osteogenesis in MSC [31]. Interestingly, adipocyte and osteoblast can be differentiated from MSC. GCs have been reported to induce lipid accumulation in 3T3-L1 cells [32]. However, as far as our knowledge, no studies report the effects of GCs on lipid accumulation in MC3T3-E1 cells. Our study showed that DEX promoted OP development, attenuated cell proliferation, and induced lipid accumulation.

Dysregulation of cholesterol metabolism exhibits a high risk for pathological development of OP [33]. Balanced cholesterol homeostasis can be maintained by cholesterol transport. ABCA1 mediates the transfer of intracellular cholesterol and phospholipids to ApoA-I to form HDL particles [34,35]. Interestingly, ABCG1 is another member of the ABC family, and it has been reported to be associated with cholesterol efflux to mature HDL independent of ApoA-I [36]. Scavenger receptor class B type I (SR-BI) is an essential mediator in regulating the transfer of cholesterol and cholesterol esters between lipoproteins and tissues [37]. SR-BI also mediates the cholesterol efflux to HDL particles from macrophages and other peripheral cells [38] and the uptake of cholesterol ester and estradiol from LDL and HDL3 in osteoblasts [39]. It has been demonstrated that DEX may decrease the expression of ABCA1, ABCG1, and SR-BI [37]. In this study, we found that DEX could increase the expression of both ABCA1 and ApoA-I in MC3T3-E1 cells. It can be postulated that DEX-induced cholesterol accumulation in MC3T3-E1 cells might be associated with the downregulation of ABCA1 expression. However, a shortcoming that the expression of HDL was not determined in this article should be further resolved.

The expression of ABCA1 is associated with the contents of cholesterol and phospholipid in the plasma membrane, affecting cell signaling transduction. The versatile roles of ABCA1 in various human diseases have been reviewed [40]. Recent evidence shows that ABCA1 has been involved in the mediation of inflammation in various diseases [41]. It has been demonstrated that ABCA1 has two potential regions for docking with and activating STAT3, which regulates the expression of the IL-6 signaling pathway [42]. Cholesterol can increase the productions of pro-inflammatory cytokines, such as TNFα, IL-6, and M-CSF in macrophages [43]. TNF-α can decrease the efflux of cholesterol in mouse osteocyte cell line MLO-Y4 by inhibiting the expression of ABCA1 [44]. Nuclear factor-κB (NF-κB) and receptor activator of NF-κB ligand (RANKL) have been implicated in osteogenic inhibition and osteoclast differentiation, promoting the development of OP [45]. Thus, it can be postulated that upregulation of ABCA1 may exhibit protective activities against OP by promoting cholesterol efflux. In this study, we found that the expression of ABCA1 was slightly up-regulated by DEX treatment. Our explanation might be that DEX caused cholesterol accumulation, which might trigger cellular stress and stimulate ABCA1 expression for homeostasis. There might be some undiscovered mechanisms associated with this issue, which still need further investigation. In addition, inhibition of ABCA1 expression was associated with decreased activity of RUNX2 and OPN in DEX-treated MC3T3-E1 cells.

GLP-1R is a class B G protein-coupled receptor (GPCR), which regulates the biological effects of GLP-1. GLP-1R is shuttled between different endomembrane compartments, mediating intracellular signaling. Once activated, GLP-1R increases insulin secretion, indicating the regulatory activity in the metabolism [46]. The downstream factors of GLP-1R include Ca^2+^/calmodulin (CaM)-dependent protein kinase (CaMK), mitogen-activated protein kinase (MAPK), PI3K/AKT, PKA, and atypical protein kinase C-ξ (PKC-ξ). These signaling pathways have been reported to regulate ABCA1 expression and cholesterol efflux. Exendin-4, an agonist of GLP-1R, has been explored to induce the expression of ABCA1 by stimulating the CaMKK/CaMKIV pathway in INS-1 cells [47]. Another study reports that the cAMP-specific inhibitor PDE4 can increase cholesterol efflux by activating ABCA1 expression in human THP-1 and mouse J774.A1 macrophages [48]. Constitutively active AKT can enhance the promoter activity of ABCA1, and the specific inhibitor PI3K LY294002 has been reported to down-regulate the expression of ABCA1 in mice [49]. In MC3T3-E1 cells, Liraglutide, an agonist of GLP-1R, has been shown to promote cell proliferation and inhibit cell apoptosis by activating the expression of PI3K/AKT and cAMP/PKA signaling pathways [50]. Our previous study found that increased expression of GLP-1R was associated with enhanced differentiation in DEX-treated MC3T3-E1 cells. In this study, the expression of GLP-1R was down-regulated in DEX-treated rats, and the activation of GLP-1R could promote cell differentiation and cholesterol accumulation inhibition by increasing ABCA1 expression in DEX-treated MC3T3-E1 cells.

GEN is an iridoid glycoside with extensive pharmacological activities. It has been shown that GEN exhibits a hypoglycemic effect in HFD mice. Recently, it has been reported that GEN increases PKA activity by modulating the activity of AMPK and AKT signaling pathways [10]. Another study is shown that GEN may ameliorate oxidative stress by increasing the expression of the cAMP/PKA/CREB pathway in PC12 cells [51]. GEN exhibits protective activity against hypoxia/reoxygenation-induced apoptosis by activating GLP-1R/PI3K/AKT signaling pathway in H9c2 cells [52]. Consistently, our previous study also showed that GEN could increase the expression of GLP-1R and promote cell differentiation in MC3T3-E1 cells. However, the molecular mechanism of GEN in increasing GLP-1R still needs further investigation. In this study, the protective activity of GEN against DEX-induced OP in vivo and in vitro by activating the expression of GLP-1R was shown. Inhibition of GLP-1R activity could abrogate the protective effects of GEN on DEX-treated MC3T3-E1 cells.

There were some limitations in this study. Our study focused on the protective activity of GEN on Dex-induced cholesterol accumulation by increasing cholesterol efflux. However, we only detected the total concentrations of the intracellular cholesterol, not the actions of cholesterol efflux. In addition, the possible molecular mechanism of GEN in mediating the expression of ABCA1 is still unclear. It is an urgent issue to explain the protective activity of GEN in lipid metabolism. ApoA-I is a major protein component of HDL in plasma, and increased apoA-I expression indicated the protective activity of GEN in lipid profile. However, the amount of HDL was not detected. ABCA1 might be responsible for free cholesterol efflux. It is difficult to directly connect the up-regulated expression of ABCA1 to the increased total intracellular cholesterol, which includes free cholesterol and cholesterol esters. More study is still needed.

## 5. Conclusions

GEN ameliorated DEX-induced differentiation inhibition and cholesterol accumulation by mediating the GLP-1R/ABCA1 axis in vivo and in vitro.

## Data Availability

The data used to support the findings of this study are included within the article.
