# Correction: Zheng et al. Geniposide Ameliorated Dexamethasone-Induced Cholesterol Accumulation in Osteoblasts by Mediating the GLP-1R/ABCA1 Axis. *Cells* 2021, *10*, 3424

**DOI:** 10.3390/cells11233838

**Published:** 2022-11-30

**Authors:** Yizhou Zheng, Yaosheng Xiao, Di Zhang, Shanshan Zhang, Jing Ouyang, Linfu Li, Weimei Shi, Rui Zhang, Hai Liu, Qi Jin, Zhixi Chen, Daohua Xu, Longhuo Wu

**Affiliations:** 1 College of Pharmacy, Gannan Medical University, Ganzhou 341000, China; yizzheng@gmu.edu.cn (Y.Z.); shanszhang@gmu.edu.cn (S.Z.); lflfllf2001@gmu.edu.cn (L.L.); wm_shi@gmu.edu.cn (W.S.); ruizhang@gmu.edu.cn (R.Z.); hailiu@gmu.edu.cn (H.L.); jinqimy@163.com (Q.J.); czxb22@163.com (Z.C.); 2 Department of Orthopedics, The First Affiliated Hospital of Gannan Medical University, Ganzhou 341000, China; yaosxiao@gmu.edu.cn; 3 Department of Medical Imaging, The First Affiliated Hospital of Gannan Medical University, Ganzhou 341000, China; dizhang@gmu.edu.cn; 4 College of Rehabilitation, Gannan Medical University, Ganzhou 341000, China; jingouy@gmu.edu.cn; 5 Key Laboratory of Traditional Chinese Medicine and New Pharmacy Development, Guangdong Medical University, Dongguan 523808, China; daohuaxu@gdmu.edu.cn

The authors wish to make the following changes to their paper [1]. Unfortunately, they made a mistake in selecting the pictures in Figure 1A (marked in red). 

Figure 1 should be changed from:


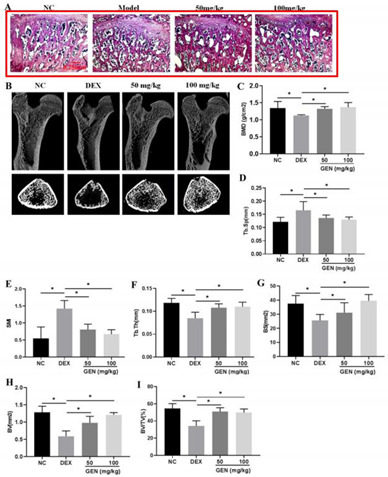


To


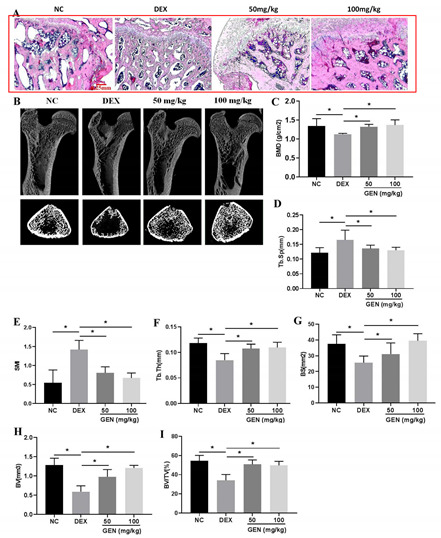


The figures in Figure 1A were totally exchanged in the four groups, i.e., the NC group, the DEX group, the DEX + 50 mg/kg group, and the DEX+100 mg/kg group. In the DEX-treated group, the bone trabecula was disordered and thinner than that in the NC group. GEN at the concentrations of 50 mg/kg and 100 mg/kg could effectively neutralize DEX-induced histopathological changes.

The changes also support the original scientific results published in *Cells* [1]. This correction was approved by the Academic Editor. The original publication has also been updated. The authors would like to apologize for any inconvenience caused to the readers by these changes.
